# Comparison of the yield of two tuberculosis screening approaches among household contacts in a community setting of Silti Zone, Central Ethiopia: a prospective cohort study

**DOI:** 10.1186/s12890-024-02950-w

**Published:** 2024-03-15

**Authors:** Habtamu Milkias Wolde, Betselot Zerihun, Waganeh Sinshaw, Delenasaw Yewhalaw, Gemeda Abebe

**Affiliations:** 1 School of Medical Laboratory Sciences, Institute of Health, Jimma University, Jimma, Oromia Ethiopia; 2 Mycobacteriology Research Center, Jimma University, Jimma, Oromia Ethiopia; 3 Tropical and Infectious Diseases Research Center, Jimma University, Jimma, Oromia Ethiopia; 4 Ethiopian Public Health Institute, Addis Ababa, Ethiopia; 5 Federal Ministry of Health, Addis Ababa, Ethiopia

**Keywords:** Household contacts, Tuberculosis, Screening, Yield, Number need to screen

## Abstract

**Background:**

Household contacts of tuberculosis (TB) patients are at a greater risk of infection and developing TB as well. Despite recommendations to actively screen such high-risk groups for TB, it is poorly implemented in Ethiopia. A community-based household contact screening was conducted to compare the yield of two different screening approaches and to identify factors associated with TB occurrence.

**Methods:**

Smear-positive pulmonary TB index cases from six health facilities in six districts of Silti Zone were identified and enrolled prospectively between September 2020 and December 2022. Trained healthcare workers conducted house visits to screen household contacts for TB. WHO (World Health Organization) recommended symptom-based screening algorithms were used. The yield of screening was compared between a two-time screening at study site I and a single baseline screening at study site II, which is the current programmatic approach. Generalized estimating equation was used to run multivariate logistic regression to identify factors associated with TB occurrence.

**Results:**

A total of 387 index TB cases (193 at site I and 194 at site II) with 1,276 eligible contacts were included for analysis. The TB yield of repeat screening approach did not show a significant difference compared to a single screening (2.3% at site I vs. 1.1% at site II, *p* < 0.072). The number needed to screen was 44 and 87 for the repeat and single screening, respectively, indicating a high TB burden in both settings. The screening algorithm for patients with comorbidities of asthma and heart failure had a 100% sensitivity, 19.1% specificity and a positive predictive value of 5.6%. Cough [AOR: 10.9, 95%CI: 2.55,46.37], fatigue [AOR: 6.1, 95%CI: 1.76,21.29], daily duration of contact with index case [AOR: 4.6, 95%CI; 1.57,13.43] and age of index cases [AOR: 0.9, 95%CI; 0.91–0.99] were associated with the occurrence of TB among household contacts.

**Conclusion:**

Our study showed that the yield of TB was not significantly different between one-time screening and repeat screening. Although repeat screening has made an addition to case notification, it should be practiced only if resources permit. Cough, fatigue, duration of contact and age of index cases were factors associated with TB. Further studies are needed to establish the association between older age and the risk of transmitting TB.

## Background

The diagnosis of an index TB case and the subsequent screening of household contacts creates an ideal opportunity for TB control programs to identify additional cases and improve case notification [1]. Prompt screening and diagnosis of TB among household contacts helps to avoid delays in treatment and the associated increased risk of mortality [2]. On the other hand, the failure of early detection and missing cases create an enormous challenge for the prevention and control of the disease. Apart from a higher risk of late diagnosis and complications, they cause an increase in the cost of care both to healthcare systems and families [2, 3]. Furthermore, the longer the missed cases stay undetected in the household, the higher the likelihood that they sustain the chain of transmission in the community. This is particularly true in places with high population density and poor living conditions, such as overcrowding and insufficient ventilation [4].

The WHO considers household contacts of TB patients as one of the high-risk groups that should be targeted by systematic active TB screening [5]. They are at an increased risk of infection and development of TB compared to the general population. The incidence of active TB in this group could be up to ten times higher than that in the general population [6]. Studies have also found a TB prevalence of up to 7.8% among household contacts of TB patients [7, 8]. Studies indicate that factors such as low income, illiteracy, smoking cigarette and HIV (Human Immunodeficiency Virus) infection are associated with TB occurrence among contacts of TB patients [9, 10]. Besides, malnutrition, previous history of TB in the family, drinking raw milk, living in a poorly ventilated and crowded houses were also factors found to be associated with TB disease in contacts of TB patients [11]. Index TB case related factors such as the anatomical site of TB being pulmonary or laryngeal, cavitation on chest radiography and close/intimate contact with family members are strongly associated with TB development among household contacts. On the other hand, age of index TB cases less than 10 years and extra pulmonary TB are associated with lower transmission as well as occurrence of TB among household contacts [12–14]. Although the WHO recommends household contact screening in high burden countries, its practical implementation is poor and faces various challenges. The absence of TB screening and diagnostic services in facilities [15], lack of adequate knowledge and skill to manage TB cases [16], scarcity of trained staff and poor screening and diagnosis practices [17] are a few of the health system-related challenges impeding its proper implementation. Fear of stigma, long distance between residence and healthcare facilities, competing priorities with screening appointments and poor knowledge about TB are some of the client-related factors affecting TB contact screening [18].

Ethiopia faces similar challenges in the implementation of household contact screening. The country introduced TB contacts register in healthcare facilities in 2014. However, various studies conducted in the country on contact screening indicate poor implementation thus far. For instance, a study conducted in the northwestern Ethiopian district of Gondar found an overall household contact screening adherence of 47.5%, indicating that more than half of eligible contacts were not screened [19]. Similarly, in another study conducted in the Tigray region of Ethiopia, only one-fifth of the index cases had at least one of their contacts screened. Only 22.6% of household contacts were screened, again indicating poor performance of household contact screening [20]. A study in five urban districts of the Amhara region in Ethiopia also showed a similar result. The proportion of adherence to contact screening was only 33.7% [21]. These findings suggest the need to devise ways to ramp up efforts to strengthen its implementation, which will help to improve the reduction of the annually missed one-third of TB cases in the country [17].

The high burden of TB in Ethiopia coupled with the low adherence to contact investigation warrants making household contact TB screening a high priority. Although household contact screening is integrated into the community-based TB prevention and control program, its effective implementation and capacity to identify cases in the Silti zone are not encouraging enough according to an unpublished zonal performance report of 2020. Hence, a prospective cohort study was conducted to estimate and compare the yield of TB among household contacts using two screening approaches and to identify individual- and household-level risk factors for TB disease in six districts of the Silti Zone in Central Ethiopia.

## Methods

### Study setting and period

Silti zone of Central Ethiopia, has 10 districts and three town administrations. The current study was conducted in six selected districts based on the reported prevalence of TB in the districts in 2019. Those districts having higher prevalence were selected for the study. The zone has a total population of 1,041,293 [22]. It is among the densely populated areas in Ethiopia, with an average of 398.4 persons per km^2^ and an average family size of 7.3 [22]. There are a total of 1,892 healthcare workers in the zone, including health extension workers (HEWs) [23]. Two hospitals and seven health centers provide TB diagnostic and treatment services in the study districts.

Community DOTS (Directly Observed Treatment Short course) implementation was started in 2010. Initial TB diagnosis and treatment initiation are performed at hospitals or nearby health centers. Patients are then sent back through referral to the health posts closest to the patients’ residence for the rest of their treatment. HEWs ensure adherence to treatment and follow-up through daily observation. However, at the second, fifth and sixth months of treatment, patients are referred back to the health centers for scheduled follow-up tests, as health posts have no TB diagnostic services [24, 25]. Apart from community DOTS, HEWs are also involved in health education, tracing treatment interrupters and loss to follow-up, identification and referral of presumptive TB cases during their routine household visits and household contact screening [24]. In 2022, the estimated prevalence of all forms of TB in the study districts ranged from 60 per 100,000 population in Kibet town to 212 per 100,000 population in Silti district [23].

This study was conducted from September 2020 to December 2022. The baseline screening was conducted from September 2020 to August, 2021 while repeat screening at study site I was conducted from September 2021 until December, 2022.

### Sample size estimation

One of the goals in this study was to compare the yield of TB cases between single baseline screening and repeat screening. The sample size is therefore calculated using a two-population proportion formula for finite populations with 5% level of significance and 1.7% margin of error. The formula for estimation of sample size for a single population proportion is:

$$n = \frac{{{{\left( {{z_{{\raise0.7ex\hbox{$\alpha $} \!\mathord{\left/{\vphantom {\alpha 2}}\right.\kern-\nulldelimiterspace}\!\lower0.7ex\hbox{$2$}}}}} \right)}^2}({p_1}{q_1} + {p_2}{q_2})}}{{{d^2}}}$$

Where:

${\text{p}}_{1}$= proportion of TB cases among household contacts with single/baseline screening

q_1_ = proportion of non-cases among household contacts with single/baseline screening.

${\text{p}}_{2}$=proportion of TB cases among household contacts with repeat screening

q_2_ = proportion of non-cases among household contacts with repeat screening.

d^2^ = desired degree of precision (margin of error).

Previous studies indicate that the proportion of TB is 1.3% among household contacts screened once at baseline [26] and 3.8% among household contacts screened at baseline and subsequently during follow up period [6]. Hence using these figures, the sample size was calculated to be 657 for each group. Including a 10% non-response rate, the total sample size became 1,445. However, as it is not possible to directly sample household contacts of TB patients, index TB cases were sampled and all eligible household members were included into the study. A previous study has shown that for every index TB case, there are a median of four contacts in a household [27]. Hence, a total of 361 index TB cases or 180 in each group are required. Since, there is only one index in a household; an index case can represent a household.

### Sampling procedure

Initially, a list of all smear positive TB cases diagnosed and registered, including those during the previous one year before the study was started, were identified from the health centers and hospitals in the study area i.e., a retrospective identification of the study participants. As the number of identified smear positive TB cases was below the estimated sample size, all of them were included at both study sites. Then, newly diagnosed smear positive TB cases were included into the study until the required sample size was obtained at both study sites. The total sample sizes were 193 at study site I and 194 at study site II. Household contacts identification was done through interviewing the index TB cases. All house household members reported by the index TB case fulfilling the household contacts operational definition were included into the study. After such a process, a total of 753 eligible household contacts were identified at study site I and 523 household contacts were found at study site II.

### Study population, the screening algorithms and the screening approaches

Smear-positive index TB cases diagnosed in healthcare facilities and their household contacts were enrolled in a prospective cohort study. The index cases were recruited from five health centers and one primary hospital providing TB diagnostic and treatment services in the study districts. Two screening approaches based on TB suggestive symptoms (cough of two weeks or more, fever, night sweating, weight loss and fatigue) were employed in this study. In three districts, i.e., study site I, both baseline and repeat screening were conducted. In the other three districts, i.e., study site II, only a single/baseline screening was performed, which is the current programmatic approach to screening in Ethiopia.

Initially, baseline screening of contacts was conducted in the households of all 397 enrolled index cases at both study sites during which general information about the study and health education on TB was provided. Then, a repeat screening of contacts was performed one year after the baseline screening among households of 193 index cases at study site I to determine whether repeat screening has a significantly higher yield.

Household contacts with signs and symptoms of TB and children aged less than five years having contact with the index case were examined for active TB through symptom screening. We used one of the WHO-recommended symptoms-based TB screening algorithms [5], which includes either a prolonged cough of two weeks or more or any two of the TB suggestive symptoms: fever of two weeks or more, night sweating, unexplained weight loss of 1.5 kg in a month and fatigue. For children under the age of 10, cough, fever, weight loss or fatigue of any duration was used. Additionally, sputum specimens from symptomatic cases were tested using GeneXpert MTB/RIF (*Mycobacterium tuberculosis*/Rifampicin) and liquid culture.

### Operational definition of variables

Systematic TB screening is defined as “the systematic identification of people with suspected active TB, in a predetermined target group, using tests, examinations and other procedures that can be applied rapidly” [5]. An index case was the first confirmed smear-positive pulmonary TB case in a household aged 16 years and older that lived with at least one other person [25]. A household contact was defined as a person who shared the same enclosed living space as the index case for one or more nights or for frequent or extended daytime periods during the three months before the start of TB treatment [28]. A presumptive TB case was a contact with cough of two weeks or more or having any two of the following symptoms: fever of two weeks or more, night sweats, and unexplained weight loss of more than 1.5 kg in a month [25]. A confirmed TB case (or simply a TB case) was a patient (household contact) from whom at least one sputum specimen taken (at baseline or during repeat screening) was positive either through the GeneXpert MTB/RIF assay or a mycobacterial culture.

### Laboratory procedures and quality control

All laboratory tests were performed at the national TB reference laboratory of Ethiopian Public Health Institute in Addis Ababa, Ethiopia. Carefully decontaminated sputum was tested using the GeneXpert MTB/RIF assay following the standard operating procedure of the manufacturer (Cephid Sunnyvale, Ca, United States) [29]. BACTEC MGIT (BD BACTEC™ MGIT™) was used for performing liquid TB culture. Processed sputum samples were inoculated into the instrument and incubated at 37 °C for a maximum of six weeks or until the instrument flagged positive. Whenever contamination was identified, the samples were reprocessed after careful decontamination following the guidance of the MGIT™ procedure manual [30]. Reagents for all procedures were checked regularly for their quality, and the instruments were checked for calibration according to the manufacturer’s instructions. Positive results of the GeneXpert test were issued to the presumptive cases through TB focal persons in the facilities as soon as results were available through phone calls. Similarly, positive culture result of study subjects whose GeneXpert results were negative were also immediately communicated to the individuals through the TB focal persons.

Data collectors were trained on how to collect and label unique IDs and store sputum samples in refrigerators before transportation. Sterile Falcon tubes were used for collecting samples. Adequacy and quality of all sputum samples were ensured before packaging and transportation to the reference laboratory. The cold chain was strictly maintained throughout the sample transportation process to keep the specimen in the temperature range of 2 to 8^0^c. Triple packaging was used, and the samples were made sure to reach the laboratory within five days of collection.

### Data collection and quality assurance

Primary data were collected through face-to-face interviews using a structured questionnaire. The questionnaire was developed in English and was then translated to Amharic, which is one of the local languages in the study districts. It was designed to gather data on households, index TB cases and their household contacts. A pretest was performed before the data collection commenced and was amended to improve its reliability among the data collectors. All data collectors were health professionals with a bachelor’s degree and were TB focal persons in their respective districts with experience in TB screening, diagnosis and treatment. All completed questionnaires were thoroughly reviewed and checked for accuracy and completeness after each data collection. Data from index TB cases residing alone and those questionnaire with incomplete household contact data were excluded from analysis.

### Data analysis

Data were entered into Microsoft Excel (Microsoft, Redmond, WA, USA) and exported to Stata version 14 (College Station Texas) for statistical analyses. The main independent variable of interest was the TB screening approach, and the outcome variable was the occurrence of TB among household contacts (yield of TB). We compared the yield of TB among households screened twice with those households screened only at baseline. Household indicators of crowding, ventilation and indoor smoke exposure were included to examine their effect on TB occurrence. The characteristics of household contacts (including characteristics of their households and index TB cases) who developed TB at baseline and later was compared to those of household contacts who did not develop TB. Frequencies and percentages were used to describe categorical variables whereas the median and interquartile range were used for all continuous variables having skewed distribution.

The traditional logistic regression model assumes the absence of correlation of risk factors or independence of observations for the model to fit well [31]. In the context of the current study, which has a hierarchical data structure, contacts living in the same household may share several risk factors, and hence, these factors could be correlated. This in turn could result in an erroneous underestimation of standard errors and higher coefficients of the independent variables. Hence, generalized estimating equations (GEEs) with robust standard errors was used to run logistic regression to account for this correlation at the household level. Predictors yielding a *p* value of < 0.20 in the bivariate analysis were included in the full model. Multicollinearity was checked, and collinear variables and others perfectly predicting the outcome variable were removed from the multivariate model. The backward variable selection method was used to build the final model. Variables having higher p values were successively removed until all the remaining variables were statistically significant at *p* < 0.05.

## Results

Initially, 199 index TB cases at site I and 198 at site II were enrolled prospectively to screen, identify and treat TB cases among their household contacts and to compare the yield of TB. Of these, 7 index TB cases (three at site I and four at site II) residing alone were excluded from the study after the initial household visit, and later, those with incomplete household contact data (*n* = 3) were excluded from analysis. In total, 387 index cases (193 at site I and 194 at site II) with 1,276 eligible household contacts were included in the analysis. A follow-up screening was also conducted one year after the baseline screening among the households of the 193 index cases (study site I) with an 80% follow-up proportion compared to the baseline screening.

### Characteristics of the households and the index TB patients

Close to 44% of the index TB cases at site I and 51% at site II lived in traditional thatched houses with a grass roof. The houses they lived in had an average area of 36 m^2^ (IQR: 24–63) at site I and 45 m^2^ (IQR: 36–70) at site II. Nearly 57.5% of the houses at site I and 54.3% at stie II had no windows at all or just one window. Approximately 17.5% of the households at site I and 42.1% at site II had a family size of six or more, but the median family size for both sites was 4 (IQR: 3–5). The majority of the households at both study sites (98% at site I and 87% at site II) used firewood or charcoal for cooking purposes. More than 73% (*n* = 142) of household heads at site I and approximately 46% (*n* = 90) at site II had no education. (Table 1)

**Table 1:** Demographic and environmental characteristics of the households of index TB patients (*n* = 193 at site I, *n* = 194 at site II), September 2020 - December 2022

Household and environmental characteristics		Study site	
		Study site I n (%)	Study site II n (%)
**Head of the household**	Wife	41 (21.2)	30 (15.5)
	Husband	136 (70.5)	161 (83.0)
	Other	16 (8.3)	3 (1.5)
**Educational status of the household head**	No education	142 (73.6)	90 (46.4)
	Primary	35 (18.1)	93 (47.9)
	Secondary	5 (2.6)	7 (3.6)
	Tertiary	11 (5.7)	4 (2.1)
**Family size**	1–5 members	155 (82.5)	165 (51.6)
	6–9 members	33 (17.5)	24 (42.1)
**House type**	Thatched houses with grass cover	85 (44.3)	99 (51.6)
	Houses with iron sheet cover	107 (55.7)	95 (49.0)
**Number of windows**	2–6 windows	82 (42.5)	84 (45.7)
	0–1 window	111 (57.5)	100 (54.3)
**House area**	>=40 m2	87 (45.3)	92 (51.7)
	< 40 m2	105 (54.7)	86 (48.3)
**Household cooking method**	Electricity	2 (1.0)	13 (6.7)
	Fire/charcoal	188 (98.0)	169 (87.6)
	Smoke free stove	2 (1.0)	11 (5.7)

All index TB patients had bacteriologically confirmed smear-positive pulmonary TB. Their median ages were 35 (IQR: 25–48) and 30 (IQR: 22–43) at sites I and II, respectively. With regards to sex, 43.3% and 46.1% of the index TB patients at site I and site II, respectively, were female. Concerning education, 60% (*n* = 111) at site I and 38% (*n* = 72) at site II had no education. The median time between the observation of the first symptom and treatment initiation of the index cases was one month at both study sites. There was a median of four and three household contacts per index case fulfilling the eligibility criteria at site I and site II, respectively. (Table 2)

**Table 2:** Sociodemographic and other characteristics of index TB patients (*n* = 193 at site I, *n* = 194 at site II), September 2020 - December 2022

Index TB case characteristics		Study site	
		Study site I n (%)	Study site II n (%)
**Age group**	<=16 years	14 (7.3)	7 (3.6)
	> 16–20 years	24 (12.6)	39 (20.2)
	> 20–40 years	87 (45.6)	96 (49.7)
	> 40 years	66 (34.6)	51 (26.4)
**Sex**	Male	107 (55.7)	104 (53.9)
	Female	85 (43.3)	89 (46.1)
**Marital status**	Single	53 (27.6)	74 (38.5)
	Married	120 (62.5)	117 (60.9)
	Divorced	11 (5.7)	0 (0)
	Widowed	8 (4.2)	1 (0.5)
**Religion**	Christian	10 (5.2)	9 (4.7)
	Muslim	182 (94.8)	184 (95.3)
**Educational status**	No education	115 (59.9)	72 (37.5)
	Primary	49 (25.5)	94 48.9)
	Secondary	18 (9.4)	19 (9.9)
	Tertiary	10 (5.2)	7 (3.7)
**First symptom to treatment initiation in days**: median (IQR)		30 (22–44)	30 (21–60)
**Body mass index**: median (IQR)		18.8 (17.3–20.5)	21.3 (18.3–24.4)
**Contacts per household**: median (IQR)		4 (3–5)	3 (2–4)

### Characteristics of household contacts

The total number of household contacts screened was 753 at site I and 523 at site II, and their median age was 23 years at both study sites. The proportions of children aged five years or younger were 4.6% (*n* = 42) and 9.4% (*n* = 48) at site I and site II, respectively. Female household contacts comprised 44.5% at site I and 47.8% at site II. In terms of educational status, 49% of the contacts at site I had no education, while this figure was 46% at site II. About 52% and 68% of the household contacts at site I and site II respectively lived in a different room from the index TB cases. (Table 3)

**Table 3:** Sociodemographic characteristics of household contacts (*n* = 753 at site I and *n* = 523 at site II), September 2020 - December 2022

Characteristics of household contacts		Study sties	
		Study site I n (%)	Study site II n (%)
**Age**	<= 5 years	42 (4.6)	48 (9.4)
	> 5–20 years	304 (40.5)	194 (37.8)
	> 20–40 years	223 (29.7)	142 (27.7)
	> 40 years	182 (24.2)	129 (25.2)
**Sex**	Male	417 (55.5)	269 (52.2)
	Female	334 (44.5)	246 (47.8)
**Marital status**	Single	387 (51.5)	272 (52.6)
	Married	306 (40.8)	230 (44.5)
	Divorced	22 (2.9)	4 (0.8)
	Widowed	36 (4.8)	11 (2.1)
**Religion**	Christian	31 (4.1)	24 (4.7)
	Muslim	720 (95.9)	490 (95.3
**Education**	No education	369 (49.2)	235 (45.5)
	Primary education	254 (33.8)	228 (44.2)
	Secondary education	94 (12.5)	46 (8.9)
	Tertiary education	34 (4.5)	7 (1.4)
**Occupation**	Government employee	74 (9.9)	19 (3.7)
	Farmer	115 (15.3)	140 (27.3)
	Merchant	91 (12.1)	45 (8.7)
	House wife	121 (16.1)	77 (14.9)
	Other	349 (46.5)	234 (45.4)
**Relationship with index**	Parent	108 (14.4)	141 (27.3)
	Spouse	123 (16.5)	78 (15.1)
	Son/Daughter	246 (32.6)	206 (39.9)
	Sibling	155 (20.6)	55 (10.7)
	Other	119 (15.9)	36 (7.0)
**Intensity of contact**	Share same bed	237 (31.6)	68 (13.1)
	Share same room	125 (16.6)	96 (18.6)
	Different room	387 (51.5)	353 (68.3)
	Other	2 (0.3)	0 (0)

Only 5% of contacts had a previous history of TB treatment at both sites, and of these, 2% reported that they either currently smoke or had a history of smoking cigarettes. Of the 188 contacts who were tested for HIV at both sites, none had an HIV infection. Among the household contacts, 16% (*n* = 117) and 10% (*n* = 52) had a cough of two weeks or more at sites I and II, respectively. Of those who reported a cough of two weeks or more, approximately 15% at site I and 7% at site II also had productive cough. On average, household contacts spent six (IQR: 6–10) and eight (IQR: 6–12) hours daily with the index TB case at sites I and II, respectively. However, children aged 5 years or younger stayed 2 more hours per day on average with the index cases. Of all the household contacts, approximately 5% (*n* = 62) had other chronic conditions, such as asthma, heart failure, hypertension, and diabetes mellitus. (Table 4)

**Table 4:** Clinical characteristics of household contacts (*n* = 753 at site I and *n* = 523 at site II), September 2020 - December 2022

Clinical characteristic of household contacts		Study sties	
		Study site I n (%)	Study site II n (%)
**Ever treated for TB**	No	721 (96.0)	489 (94.8)
	Yes	30 (4.0)	27 (5.2)
**Smoking status**	No	732 (97.5)	507 (99.0)
	Yes	19 (2.5)	5 (1.0)
**BCG vaccination status**	Scar present	53 (7.1)	251 (48.7)
	Certificate of vaccination	27 (3.6)	24 (4.7)
	Vaccine recorded, no scar	163 (21.7)	70 (13.6)
	Not vaccinated	508 (67.6)	170 (33.0)
**Recently, tested for HIV**	No	679 (90.4)	386 (74.7)
	Yes	72 (9.6)	131 (25.3)
**HIV status**	Negative	40 (100.0)	134 (100.0)
	Positive	0 (0.0)	0 (0.0)
**Cough > 2 weeks**	No	634 (84.4)	465 (89.9)
	Yes	117 (15.6)	52 (10.1)
**Productive cough**	No	638 (84.9)	480 (92.8)
	Yes	113 (15.1)	37 (7.2)
**Blood mixed sputum**	No	739 (98.4)	514 (99.4)
	Yes	12 (1.6)	3 (0.6)
**Fever**	No	716 (95.3)	493 (95.4)
	Yes	35 (4.7)	24 (4.6)
**Night sweating**	No	689 (91.7)	500 (96.7)
	Yes	62 (8.3)	17 (3.3)
**Appetite loss**	No	697 (92.8)	494 (95.6)
	Yes	54 (7.2)	23 (4.5)
**Weight loss**	No	716 (95.3)	498 (96.3)
	Yes	35 (4.7)	19 (3.7)
**Chest pain**	No	718 (95.6)	500 (96.7)
	Yes	33 (4.4)	17 (3.3)
**Fatigue**	No	676 (90.0)	499 (96.9)
	Yes	75 (10.0)	16 (3.1)
**Other comorbid illness**	No	714 (95.1)	481 (95.1)
	Yes	37 (4.9)	25 (4.9)
**BMI**: median (IQR)		19.5 (17.5–21.9)	21.4 (18.3–23.4)
**Daily contact duration in hrs**.: median (IQR)		6 (6–10)	8 (6–12)
**Total contact duration in days**: median (IQR)		30 (20–60)	120 (30–365)

### Comparison of the yield of household contact TB screening

A total of 98 (13%) presumptive TB cases were identified from 753 screened household contacts at site I. Similarly, 65 (12%) presumptive TB cases were identified from 523 screened household contacts at site II during baseline screening. Additionally, 55 (9.4%) presumptive TB cases were found among 585 household contacts during repeat screening at site I. Approximately 70% of presumptive TB cases (*n* = 38) identified during repeat screening were found in households where presumptive cases were found during baseline screening.

Eleven TB cases of all forms (1.5%) were identified during baseline screening at site (I) Six more TB cases (1%) were also diagnosed at site I during repeat screening a year later. At site II, where the current programmatic screening approach was employed, six TB cases of all forms (1.1%) were found during baseline screening. The overall yield of TB identified at site I was 2,258 per 100,000 (95% CI: 1,407–3,604), and it was 1,149 per 100,000 (95% CI: 517–2,538) at site (II) There was no statistically significant difference in the yield of TB cases identified at the two sites. Altogether, the household contact screening yielded a total of 23 (1.8%) TB cases of all forms at both study sites.

The number needed to screen (NNS) values for presumptive TB cases was 6 and 12 for sites I and II, respectively. Similarly, the NNS for finding a single TB case were 44 and 87 for sites I and II, respectively. On the other hand, the number needed to test (NNT), i.e., the number of presumptive TB cases that needed to be tested to identify one case of TB, was 9 and 11 for sites I and II, respectively. (Table 5)

**Table 5:** Comparison of yield of household contact screening between two screening approaches; September 2020 - December 2022

Household contacts TB screening	Screening (both groups) n (%)	Study site I (repeat screening) n (%)	Study site II (single baseline screening) n (%)
Index TB cases screened	387	193 (49.9)	194 (50.1)
Household contacts screened at baseline	1,276	753 (59.1)	523 (40.9)
Contacts screened during repeat screening	585	585	NA
Presumptive TB cases at baseline	163 (12.7)	98 (13)	65 (12)
Presumptive TB cases at repeat screening	55 (9.4)	55 (9.4)	NA
TB yield during baseline screening	17 (1.3)	11 (1.5)	6 (1.1)
TB yield during repeat screening	6 (1)	6 (1)	NA
TB yield (total)	23 (1.8)	17 (2.2)	6 (1.1)
NNS value for presumptive TB cases	6	6	12
NNS value for TB cases	55	44	87
NNT value for TB cases	9	9	11

NA = not applicable; NNS = number needed to screen; NNT = number needed to test

All identified TB patients were treated based on the national TB treatment guidelines with the first-line anti-TB regimen for six months, as none of the cases were resistant to rifampicin according to the GeneXpert test. Of the eligible under five children, 48% (*n* = 43) were initiated on TB preventive therapy after clinical evaluation to exclude TB disease.

### Sensitivity and specificity of the TB screening algorithm among household contacts with chronic conditions

Among the total of 62 household contacts with comorbid illnesses, 22 (35%) patients were found to have either an asthma or a heart failure diagnosis, and most had a chronic cough of more than two weeks. Among these patients, 18 (82%) were identified as presumptive TB cases and were requested to provide sputum for testing. Only one patient was found to have TB. The screening algorithm had 100% sensitivity, but the specificity was only 19.1%, and the positive predictive value was 5.6%. (Table 6)

**Table 6:** Evaluation of the screening algorithm* for household contacts with comorbid (Asthma and Heart Failure) illnesses

Screening test result	TB disease (TB Culture)		Total
	Present	Absent	
Positive	1	17	18
Negative	0	4	4
Total	1	21	22

*Screening algorithm for adults and children > = 10 years: prolonged/chronic cough of two weeks or more or any two of the TB suggestive symptoms: fever of two weeks or more, night sweating, unexplained weight loss of 1.5 kg in a month and fatigue.*

### Factors associated with TB among household contacts

Generalized estimating equation (GEE) results show that the odds of finding a TB case among the household contacts were similar between the two screening approaches at the bivariate level [crude odds ratio (COR): 2, 95% CI; 0.79–5.02]. The odds of finding TB were still the same between the two screening approaches even after adjusting for other variables [adjusted odds ratio (AOR): 0.9, 95% CI; 0.29,2.91]. Other household-level variables with no association with the occurrence of TB were family size, education level of the household head and cooking method used. Index case characteristics such as sex, BMI and duration between first symptom and treatment also had no association with the outcome variable. The characteristics of household contacts, including age, sex, education level, occupation, smoking status and presence of comorbid chronic illnesses, were not associated with the occurrence of TB and were not selected for inclusion in the multivariate model.

On bivariate logistic regression using GEE, household contacts with reported clinical symptoms such as chronic cough of two weeks or more [COR: 35, 95%CI; 11.7–104.0], observation blood mixed sputum [COR: 16, 95%CI; 4.32–64.53], fever [COR: 9.9, 95%CI; 3.91–25.22], night sweating [AOR: 8.9 95%CI; 3.67–21.69], loss of appetite [COR: 9, 95%CI: 3.72,22.15], weight loss [COR: 11.4, 95%CI; 4.46,28.96], chest pain [COR: 5.5, 95%CI; 1.80,16.79] and fatigue [COR: 29.3, 95%CI: 12.08,71.28] were found to have association with occurrence of TB. However, only chronic cough and fatigue were found to be independent predictors among the symptoms on multivariate regression. Accordingly, contacts with chronic cough had nearly 11 times higher odds of developing TB than those without symptoms after adjusting for other variables [AOR: 10.9, 95% CI: 2.55,46.37]. Household contacts who reported chronic fatigue were also 6 times as likely to develop TB as household contacts without the symptom [AOR: 6.1, 95% CI: 1.76,21.29] after controlling for cough, daily duration of contact and age of household contacts. (Table 7)

**Table 7:** Environmental- and individual-level factors associated with TB among household contacts (*n* = 1,276), September 2020 - December 2022

Household and individual factors		TB status		COR [95%CI]	AOR [95%CI]
		No (%)	Yes (%)		
Screening approach	Baseline only	516 (98.9)	6 (1.1)	1	1
	Repeat screen	736 (97.7)	17 (2.3)	2 [0.79,5.02]	0.9 [0.29,2.91]
House area	40–717 m^2^	556 (97.5)	14 (2.5)	1	1
	20–40 m^2^	656 (98.6)	9 (1.4)	0.5 [0.24,1.24]	0.4 [0.15,1.12]
Electricity use	No	574 (96.6)	14 (2.4)	1	1
	Yes	677 (98.7)	9 (1.3)	0.6 [0.24,1.26]	0.6 [0.19,1.72]
Marital status of index cases	Single	385 (98.2)	7 (1.8)	1	1
	Married	786 (98.5)	12 (0.15)	0.8 [0.33,2.10]	1.5 [0.42,49.7]
	Divorced	41 (95.4)	2 (4.7)	2.6 [0.54,12.81]	4.23 [0.65,27.43]
	Widowed	34 (94.4)	2 (5.6)	3.3 [0.67,15.75]	26.9 [2.26,320.71]
BMI of contacts	> 20	578 (98.8)	7 (1.2)	1	1
	<=20	573 (97.3)	16 (2.7)	2.3 [0.94,5.63]	1.5 [0.56,4.24]
Cough	No	1,095 (99.6)	4 (0.4)	1	1
	Yes	149 (88.7)	19 (11.3)	35 ]11.7,104.0]	10.9 [2.55,46.37] *
Blood in sputum	No	1,233 (98.4)	20 91.6)	1	1
	Yes	11 (78.6)	3 (21.4)	16 [4.32,64.53]	3.3 [0.69,15.83]
Fever	No	1,192 (98.7)	16 (1.3)	1	1
	Yes	52 (88.1)	7 (11.9)	9.9 [3.91,25.22]	0.9 [0.33,2.98]
Night sweating	No	1,173 (98.7)	15 (1.3)	1	1
	Yes	71 (89.9)	8 (10.1)	8.9 [3.67,21.69]	0.5 [0.14,1.61]
Loss of appetite	No	1,175 (98.7)	15 (1.3)	1	1
	Yes	69 (89.6)	8 (10.4)	9 [3.72,22.15]	0.4 [0.09,1.96]
Weight loss	No	1,198 (98.7)	16 (1.3)	1	1
	Yes	46 (86.8)	7 (13.20	11.4 [4.46,28.96]	2.5 [0.53,11.64]
Chest pain	No	1,198 (98.4)	19 (1.6)	1	1
	Yes	46 (92.0)	4 (8.0)	5.5 [1.80,16.79]	0.5 [0.15,1.95]
Chronic fatigue	No	1,167 (99.3)	8 (0.7)	1	1
	Yes	75 (8.3)	15 (16.7)	29.3 [12.08,71.28]	6.1 [1.76,21.29] *
Contact duration before index case treatment	> 37 days	622 (8.9)	7 (1.1)	1	1
	<= 37 days	606 (97.4)	16 (2.6)	2.2 [0.45,5.56]	2.1 [0.71,6.15]
Daily duration of contact	< 8 h.	778 (99.2)	6 (0.8)	1	1
	>=8 h.	399 (95.9)	17 (4.1)	5.5 [2.16,13.93]	4.6 [1.57,13.43] *
Age of index cases				0.9 [0.96,1.02]	0.9 [0.91,0.99] *

*: significant at *p* < 0.05

Household contacts with more than eight hours of daily contact with index TB cases had 4.6 times higher odds of developing TB than those who had eight hours or less of daily contact after adjusting for the other variables in the model [AOR: 4.6, 95% CI; 1.57,13.43]. The age of the index cases was also found to have a statistically significant association with TB occurrence among household contacts. An increase in the age of an index case is associated with lower odds of developing TB among household contacts [AOR: 0.9, 95% CI; 0.91–0.99]. (Table 7).

## Discussion

In this study, a third (35%) of the households had at least one presumptive TB case and their prevalence was 18%. An overall yield of 1.8% (all forms) of TB was found among the household contacts. There was no significant difference in the yield of TB between one time screening and repeat screening. The study also showed that screening algorithms involving chronic cough symptom for patients with comorbidities such as Asthma and Heart failure had a high sensitivity but a very low specificity (19.1%) and positive predictive value (5.6%). The logistic regression model using GEE revealed chronic cough, fatigue, daily duration of contact and the age of index cases as independent predictors of TB occurrence among the household contacts.

This study found no significant difference in the yield of TB between the two screening approaches (i.e., a single baseline screening vs. baseline plus repeat screening after a year), with the proportions of TB identified being 1,149 per 100,000 household contacts and 2,258 per 100,000 household contacts. This indicates that the current one-time screening programmatic approach was performing almost as effectively as a two-time screening. Most of the cases were found during baseline screening as prevalent cases at both study sites. On the other hand, repeat screening after a year found six additional cases with an incidence rate of 1,026 per 100,000 contacts, a finding falling in the range found in other studies [7, 32]. Finding fewer cases during repeat screening may be partly ascribed to loss to follow-up or decreased proportion of attendance during subsequent screens [33], which could also be the case in our study. Repeated screening requires more time and resources. The WHO suggests that repeat screening be carried out only when resources permit [5]. Hence, for resource-constrained settings such as Ethiopia, one-time screening of contacts could be as effective if managed properly. However, studies consistently indicate that the incidence of TB continues for at least two years after initial exposure [32, 34, 35] and hence the importance of repeated screening.

The number needed to screen is an approximate indicator of the cost effectiveness of a TB screening program [5]. As the prevalence of undetected TB in the population increases, the NNS tends to decrease. Both the NNS and NNT are usually lower in high TB burden settings [5]. Studies indicate that an average of seven presumptive TB cases need to be screened to identify one case of TB [36, 37]. In a high burden and resource-poor countries, targeting key populations such as household contact screening is beneficial in terms of resource efficiency, as the number of people required to be screened and tested significantly lowers. In our study, the NNS was found to be 55, which is slightly lower but lies within a range reported by a study conducted in both facility and community settings in Ethiopia [26]. Our finding also falls inside the range of NNS which is between 34 and 79 reported by other studies conducted on high TB risk groups [26, 38, 39].

The screening algorithm used was highly sensitive (100%) among patients with comorbid asthma and heart failure. However, only one case of TB was found among these patients, indicating poor specificity and positive predictive value (PPV). The possible explanation for this is the fact that both conditions usually cause chronic cough [40], and this might be inaccurately considered a TB suggestive symptom whenever these patients become contacts of a TB patient. A low PPV indicates that a positive screening test result is likely to be incorrect, and hence, the screening test performs poorly compared to the gold standard test [41]. Thus, the screening algorithm, which includes chronic cough, might not be appropriate for such cases, and there is a need to devise other screening methods with better performance, such as chest X-ray.

In this study, no household-level factor was associated with TB occurrence among household contacts. Nonetheless, all of the TB cases identified occurred in households with their heads having either no or primary education. Studies indicate that lower education results in delayed TB diagnosis [42], which in turn causes more transmission. Among the index TB case characteristics, age was found to have an association with TB occurrence. The older the index cases are, the lower the chances of finding TB cases among their contacts. This is in agreement with the fact that although they are at higher risk of acquiring TB [43–45], the frequency and intensity of coughing decreases by limiting transmission [46]. It could also be possible that the older index cases might spend less time with their contacts. Household contact characteristics such as chronic cough, fatigue and daily duration of contact showed a significant association with TB occurrence among household contacts. Studies consistently confirm that these factors are strongly associated with TB occurrence among contacts of TB patients [40, 47–49].

One of the limitations of this study is that sputum was obtained from a few children aged less than ten years. Children are less likely to provide spontaneous sputum for testing than adults [50]. However, sputum induction could have been considered, and this might have had an impact on the yield of TB. Additionally, the absence of other screening methods, such as chest X-ray and adjunct clinical diagnosis, may have also caused some cases to be missed, hence resulting in underestimation of the yield. Furthermore, molecular tests and phylogenetic analyses were not done to describe the similarities in the identities of the strains from the index TB cases and the secondary cases. However, this is the first community-based prospective study that attempted to compare the yield of two different screening approaches in a mostly rural setting by employing better performing diagnostic tests.

## Conclusion

Our study demonstrated that there was no significant difference between one-time vs. two-time screening in the yield of TB. The repeat screening captured additional cases and was an enormous addition to the case notification but should be practiced only if resources are sufficiently available. The TB program in the zone should therefore, ensure that all household contacts are screened at least once. Cough, fatigue, duration of contact and age of the index cases were factors associated with TB occurrence among household contacts. Cough-based screening algorithms have a high sensitivity but a very low positive predictive value among household contacts having a concurrent chronic illness with cough, such as asthma and heart failure. Hence, alternative screening methods should be applied for improved positive predictive value. Further studies are needed to establish the association between older age and the risk of transmitting TB.

## Data Availability

The raw dataset of this study can be obtained from the corresponding author upon reasonable request.

## Abbreviations

AOR: Adjusted Odds Ratio
CI: Confidence Interval
COR: Crude Odds Ratio
EPHI: Ethiopian Public Health Institute
EPTB: Extrapulmonary Tuberculosis
GEE: Generalized Estimating Equations
HEW: Health Extension Worker
HIV: Human Immuno-deficiency Virus
IQR: Inter Quartile Range
NNS: Number Needed to Screen
NNT: Number Needed to Test
RHB: Regional Health Bureau
TB: Tuberculosis
WHO: World Health Organization
